# Cumulative asbestos exposure as a key predictor of long-term pleuropulmonary outcomes: insights from decades of follow-up

**DOI:** 10.1007/s00420-025-02143-w

**Published:** 2025-05-22

**Authors:** Ladislav Štěpánek, Marie Nakládalová, Magdaléna Janošíková, Lubomír Štěpánek, Alena Boriková

**Affiliations:** 1 Department of Occupational Medicine, University Hospital Olomouc and Faculty of Medicine and Dentistry, Palacký University Olomouc, Zdravotníků 248/7, Olomouc, 77900 Czech Republic; 2 Institute of Biophysics and Informatics, First Faculty of Medicine, Charles University, Salmovská 1, Prague, 12000 Czech Republic

**Keywords:** Asbestos, Exposure, Latency, Pleural mesothelioma, Radiological changes

## Abstract

**Purpose:**

Occupational asbestos exposure was widespread before regulatory bans, and it remains a risk during renovations or demolitions of older buildings. While asbestos-related diseases are well-documented, less is known about minor radiological changes in exposed individuals. This longitudinal study aimed to identify predictors of pleural and parenchymal lung disorders in individuals with previous occupational asbestos exposure, focusing on both established asbestos-related diseases and minor radiological abnormalities.

**Methods:**

The study tracked 445 former employees (334 men, 111 women) of two Czech asbestos-processing plants, who underwent regular examinations from the 1980s to December 2022. Cox proportional hazards regression models were employed to analyse predictors of asbestos-related diseases, as well as minor radiological findings alone.

**Results:**

Over a median latency of 37 years, 127 participants (28.5%) developed asbestos-related diseases, mainly pleural mesothelioma (59 cases). An additional 168 participants (37.8%) exhibited minor radiological findings, predominantly pleural plaques (129 cases), while 150 (33.7%) had no abnormalities. Substantial cumulative exposure was a strong predictor for minor radiological findings (odds ratio [OR] 1.98, 95% confidence interval [CI] 1.18–3.35, *p* = 0.010) and any endpoint, including diseases (OR 1.89, 95% CI 1.18–3.02, *p* = 0.008). Respiratory symptoms and impaired spirometry results significantly increased the likelihood of endpoint occurrence. No significant differences emerged between settings with predominantly chrysotile exposure and those with a chrysotile-crocidolite mixture.

**Conclusion:**

This study highlights the predictive value of cumulative exposure and the need for ongoing surveillance of occupationally exposed individuals to better understand radiological changes, their significance, and to refine risk assessment models.

## Introduction

Asbestos is a collective term applied for the commercial identification of a heterogeneous group of minerals whose crystals form bundles of easily separable fibres. These minerals derive from eruptive metamorphic rocks that, due to a natural process of recrystallization, form a fibrous material. They belong to two different groups: (a) amphiboles, which include amosite (brown asbestos), crocidolite (blue asbestos), anthophyllite, actinolite, and tremolite; and (b) serpentines, represented by chrysotile (white asbestos) (Vicari et al. 2023).

Asbestos, prized for its unique properties such as resistance to heat, cold, electricity, and chemicals, saw widespread use beginning in the late 19th century, peaking in the mid-20th century. Its versatility led to the incorporation into thousands of products, predominantly in construction materials, including asbestos cement, as well as in shipbuilding and various household items. Globally, the majority of asbestos was used in the production of construction materials (Frank et al. 2024).

In 1973 the International Agency for Research on Cancer declared asbestos in all its commercial forms as an established human carcinogen (IARC 2012). Consequently, partial national policies restricted or completely banned the use of asbestos and the production of asbestos-containing items. The progressive ban on the use of asbestos in the European Union (EU) began in 1988 with the prohibition of crocidolite and was subsequently extended to cover other asbestos-containing materials (ACMs). Since 2005, all forms of asbestos have been banned in the EU. Nonetheless, asbestos is still part of the daily life of the population as ACMs are still present in many buildings constructed and renovated before the end of the 1990s (Council of the EU 2009).

While strict regulations in the Western world have minimized current asbestos exposure, the latent nature of its pathophysiological effects means that the legacy of past exposures, particularly from industries like mining and asbestos-cement manufacturing, continues to impact many individuals. Moreover, workers engaged in the renovation or demolition of older buildings remain at risk of potentially significant exposure. Non-occupational exposure can also occur, particularly during home renovations or through incidental handling of ACMs (Frank et al. 2024).

The adverse effects of asbestos generally fall under 3 categories: pleural disease, lung parenchymal disease, and neoplastic disease elsewhere (Norbet et al. 2015). Asbestos-induced carcinogenesis is a complex event resulting from different factors, including physicochemical characteristics of the fibres (dimension, surface reactivity and chemical composition), intensity and duration of exposure and, finally, host genetic determinants. Although asbestos damages primarily the airways, its deleterious malignant effect has been proven in the peritoneum, ovaries, testes and gastrointestinal tract. While asbestos-related malignancies are well recognized, the predictors and role of non-malignant radiological changes remain underexplored (Zunarelli et al. 2021; Thives et al. 2022).

This longitudinal study aimed to identify potential predictors of pleural and parenchymal lung diseases in individuals with a history of occupational asbestos exposure. Particular attention was given to radiological abnormalities in the chest, including those that do not meet the diagnostic criteria for asbestos-related diseases.

## Methods

### Study population

The study participants were recruited from individuals attending regular follow-ups (biennial or more frequent) at the Department of Occupational Medicine, University Hospital Olomouc, Czech Republic. These follow-ups arose from (1) post-exposure examinations, mandated by Czech legislation, for former employees with documented occupational asbestos exposure. These lifelong examinations, conducted at two-year intervals, included updates of medical history, chest radiography, spirometry, and other diagnostic tests aimed at the early detection of asbestos-related conditions (Decree No. 79/2013). (2) Referral-based evaluations for suspected occupational diseases caused by asbestos, initiated when patients were referred from other clinical departments, often for assessment of work-related mesothelioma or other pathologies. Recruitment commenced in 1980, with most post-exposure follow-ups beginning in the first two decades. Additional participants were gradually incorporated into the surveillance program over subsequent years.

For this study, individuals were included if they had attended examinations consistently until either the development of a severe health condition precluding further follow-ups or a personal decision to discontinue. Data collection encompassed all follow-up visits conducted up to December 31, 2022. The sole exclusion criterion was insufficient records of occupational asbestos exposure history to ensure the reliability of exposure classification. This primarily applied to workers with unclear job assignments due to rotation across multiple production processes or insufficient documentation on task durations, which were crucial for assessing exposure-related parameters.

The sample for this longitudinal study comprised 445 individuals, all of whom were former employees of two asbestos-processing plants in the Olomouc region, Czech Republic. The first plant (Plant A, *n* = 255) operated between 1959 and 1993 and manufactured asbestos-cement products (pipes and boards) from a mixture of 20% crocidolite and 80% chrysotile. The second plant (Plant B, *n* = 190) operated from 1910 to 1995 and produced asbestos-cement corrugated roofing sheets using asbestos containing up to 4% crocidolite until 1978, after which only chrysotile was used. The plants differed in their spatial layout: Plant A operated within a single undivided production hall, whereas Plant B had separate sections for different stages of production. However, historical workplace assessments indicated that dust levels in both plants were comparable.

### Collected data

Endpoints reflecting radiological changes in the pleura and lungs were categorized into three subgroups for comparative analysis:

1. Individuals who developed asbestos-related occupational diseases listed in Czech legislation (Government Reg. No. 290/1995), including non-malignant respiratory conditions such as hyalinosis, i.e. pleural plaques (PP) with restrictive ventilatory impairment, asbestosis, i.e. asbestos-related interstitial fibrosis with at least s 2/2, 12/2 or u 2/2 changes according to the International Labour Organization (ILO) classification - Classification for B Readers (ILO 2022, NIOSH 2024), pleural mesothelioma, or asbestos-associated bronchogenic carcinoma (accompanied by hyalinosis or asbestosis with at least s 1/1, 11/1 or u 1/1 changes according to the ILO classification);
2. Individuals presenting minor radiological findings indicative of early fibrotic changes in the pleura or lungs that did not meet the criteria for asbestos-related diseases from (1). Three types of pleural abnormalities were recognized: PPs (localized pleural thickening), costophrenic angle obliteration, and diffuse pleural thickening or calcification (ILO 2022). Parenchymal lung changes observed in individuals with pleural abnormalities were also recorded;
3. Individuals without any radiological endpoints from (1) or (2), for whom data from their last follow-up examination were considered.

The following data were collected from individuals in the study sample:

- Exposure duration: Measured as the total number of years each individual worked in environments with asbestos dust exposure.
- Dust levels: Classified as *low* or *high*, based on the subject’s specific occupational role within the production process and historical hygiene assessments conducted in both plants. These assessments utilized a gravimetric method (measuring mg/m^3^) and a membrane filter method (measuring fibres/cm^3^). High dust levels, with regularly exceeded permissible exposure limits, were identified in Plant A at workstations associated with raw material processing, asbestos milling and fiberizing, board cutting, and pipe manufacturing. Similarly, in Plant B, high dust levels were recorded in areas dedicated to raw material processing, roller mills, fiberizers, primary production, and cutting operations.
- Cumulative exposure: Combined exposure duration and dust levels. For statistical analysis, two categories were defined:
  - *Mild*: Less than 5 years of exposure to *high* dust levels or exclusively *low* dust level exposure.
  - *Substantial*: At least 5 years of exposure to *high* dust levels.
- Latency: Defined as the interval between the onset of exposure and the development of an endpoint.
- Imaging results: Chest skiagrams obtained during routine check-ups, with (positron emission tomography/)computed tomography ([PET/]/CT) performed when clinically indicated.
- Smoking status: Categorized as current smoker, ex-smoker, or non-smoker.
- Spirometry results: Classified as normal, mild, moderate, or severe impairment. Results were determined by reductions in forced expiratory volume in the first second (for obstructive disorders) or functional vital capacity (for restrictive disorders) below thresholds of 80%, 60%, or 45% of predicted values (Kociánová 2017). Measurements were accounted for at the time of the endpoint.
- Symptoms: Respiratory complaints such as dyspnea on exertion or at rest, cough, wheezing, or chest pain, documented at the endpoint.

### Statistics

Statistical analyses were conducted in the R software environment (R Foundation for Statistical Computing; http://www.r-project.org/). All numerical variables were characterized with descriptive statistics. The normality of the variables was assessed using the Shapiro-Wilk test. The significance of differences between groups was determined using the Student’s t-test for continuous variables with a normal distribution, the Mann-Whitney test for asymmetrically distributed continuous variables, and the chi-square test for categorical variables. To explore the relationship between multiple independent variables and a composite dependent variable, Cox proportional hazards regression with right censoring was applied. The dependent variable was defined by endpoint occurrence and latency or endpoint-free period in those without endpoints. Two regression models were used: one to predict any endpoint and the other to predict minor radiological findings exclusively. Kaplan-Meier survival curves were constructed to visualize the time-to-event distribution, with latency (years) on the x-axis and the proportion of endpoint-free individuals on the y-axis. The significance level was set at 5%.

## Results

The study sample comprised 445 individuals (111 women and 334 men) with a mean age of 62.3 years (median 62) at the time of the endpoint or the most recent examination. Participants had an average duration of asbestos exposure of 14.6 years (median 13), with high dust levels reported for 77.3% of the cohort and substantial cumulative exposure for 60.7%. The mean latency period to the endpoint was 37.4 years (median 37). Non-smoking individuals accounted for 63.4% of the cohort, while 36.6% had a history of smoking or were active smokers during the study period.

By the end of the follow-up period, 127 individuals (28.5%) had developed asbestos-related diseases, 168 individuals (37.8%) exhibited only minor radiological findings, and the remaining 150 individuals (33.7%) showed no detectable abnormalities. Significant differences were observed between these subgroups in several characteristics (Table 1). Individuals with asbestos-related diseases were slightly older than those in other subgroups. While the distribution of dust levels was comparable across subgroups, those who developed diseases or radiological findings had significantly longer exposure durations. This pattern was reflected in a descending gradient of substantial cumulative exposure from individuals with diseases to those with minor radiological findings and, finally, to individuals without abnormalities. A total of 181 individuals (61.4%) who developed any endpoint had exposure durations equal to or exceeding the median (≥ 13 years), whereas 105 individuals (70,0%) without an endpoint were exposed for shorter periods than the median (*p* < 0.001). The latency or the interval between the start of exposure and the last examination in cases without an endpoint did not differ significantly between subgroups. Non-smokers were most prevalent among those who did not develop any endpoints. Spirometry also displayed significant differences, with a higher frequency of normal results and a lower frequency of abnormal results among individuals without an endpoint. An unfavourable gradient was evident from those without radiological abnormalities to participants with diseases. Similarly, respiratory symptoms were most common in individuals with diseases, with nearly all (95.3%) symptomatic.

**Table 1:** Characteristics of the study population *p*-values indicate the statistical significance of differences between adjacent subgroups (columns)

Characteristics		Entire sample	Subgroups				
			Asbestos-associated diseases	Minor radiological findings	No endpoint	Asbestos-assoc. dis. vs. minor radiol. findings, *p*-value	Minor radiol. findings vs. no endpoint, *p*-value
N		445	127	168	150		
Age at the endpoint (years)		62.3 (61.4; 63.1)	64.1 (62.4; 65.9)	60.9 (59.6; 62.2)	62.2 (60.7; 63.7)	0.004	0.200
Sex (N, %)	females	111 (24.9)	34 (26.8)	41 (24.4)	36 (24.0)	0.644	0.933
	males	334 (75.1)	93 (73.2)	127 (75.6)	114 (76.0)		
Exposure duration (years)		14.6 (13.7; 15.6)	20.5 (18.7; 22.4)	14.0 (12.5; 15.5)	10.3 (8.9; 11.8)	< 0.001	< 0.001
Dust level (N, %)	low	101 (22.7)	23 (18.1)	34 (20.2)	44 (29.3)	0.647	0.060
	high	344 (77.3)	104 (81.9)	134 (79.8)	106 (70.7)		
Cumulative exposure (N, %)	mild	175 (39.3)	33 (26.0)	60 (35.7)	82 (54.7)	0.075	0.001
	substantial	270 (60.7)	94 (74.0)	108 (64.3)	68 (45.3)		
Latency/*follow-up* (years)		37.4 (36.4; 38.3)	38.3 (36.3; 40.3)	36.8 (35.3; 38.3)	*37.2 (35.7; 38.6)*	0.244	0.936
Place of exposure (N, %)	Plant A	255 (57.3)	81 (63.8)	98 (58.3)	76 (50.7)	0.343	0.170
	Plant B	190 (42.7)	46 (36.2)	70 (41.7)	74 (49.3)		
Smoking status (N, %)	non-smoker	282 (63.4)	77 (60.6)	104 (61.9)	101 (67.3)	0.971	0.018
	ex-smoker	64 (14.4)	23 (18.1)	30 (17.9)	11 (7.3)		
	smoker	99 (22.2)	27 (21.3)	34 (20.2)	38 (25.3)		
Spirometry (N, %)	normal	253 (56.9)	16 (12.6)	110 (65.5)	127 (84.7)	< 0.001	0.002
	mild	101 (22.7)	35 (27.6)	46 (27.4)	20 (13.3)		
	moderate	17 (3.8)	8 (6.3)	7 (4.2)	2 (1.3)		
	severe	12 (2.7)	8 (6.3)	4 (2.4)	0		
Symptoms (N, %)	present	249 (56.0)	121 (95.3)	78 (46.4)	50 (33.3)	< 0.001	0.017
	absent	196 (44.0)	6 (4.7)	90 (53.6)	100 (66.7)		

Among individuals with asbestos-related diseases, the most common diagnoses were pleural mesothelioma (59 cases, 46.5%), hyalinosis (32 cases, 25.2%), lung cancer (21 cases, 16.5%), and asbestosis (19 cases, 15.0%), with four individuals developing two different diseases over time. In the subgroup with only minor radiological changes, the most frequent findings on chest skiagrams were PPs (51 cases, 30.4%), pulmonary nodules (33 cases, 19.6%), reticular interstitial patterns (32 cases, 19.0%), linear pulmonary shadows (17 cases, 10.1%), and fibrotic changes in the lungs (8 cases, 4.8%). A total of 143 individuals (85.1%) in this subgroup underwent (PET/)CT scans, which revealed the most common findings as PPs (129 cases, 76.8%), pulmonary nodules (61 cases, 36.3%), fibrotic changes in the lungs (39 cases, 23.2%), ground-glass opacities (25 cases, 14.9%), reticular interstitial patterns (8 cases, 4.8%), and focal lung lesions (5 cases, 3.0%).

The entire cohort accumulated 9,685 person-years of follow-up (5,530 in Plant A and 4,155 in Plant B), calculated from the first medical evaluation to the last follow-up or disease onset, whichever came first. Nearly half of the sample (194 individuals, 43.6%) had a follow-up examination within the last 2 years of the study period. This included 6 individuals with an asbestos-related disease, 107 with minor radiological changes, and 81 without any endpoint. No significant difference was found in the follow-up rates between those with minor changes (63.7%) and those without an endpoint (54.0%, *p* = 0.373).

The regression model identified several significant predictors for the development of any endpoint (Table 2). For each additional year of age, the odds of developing asbestos-related diseases or minor radiological changes decreased by 0.83 times. Similarly, for each additional year of exposure, the odds of an endpoint decreased by 0.98 times. Substantial cumulative exposure increased the likelihood of an endpoint by 1.89 times compared to mild exposure. Individuals with mild (odds ratio [OR] 1.35) or severe (OR 1.93) ventilation impairment had significantly higher odds of an endpoint compared to those with normal spirometry results. Perceived symptoms at the time of examination also significantly predicted the likelihood of an endpoint (OR 1.40). The second regression model, focusing exclusively on minor radiological findings, identified three significant determinants. Each additional year of age reduced the odds of radiological changes by 0.82 times, and each year of exposure decreased the odds by 0.97 times. In contrast, substantial cumulative exposure increased the odds of developing radiological changes by 1.98 times compared to mild exposure.

**Table 2:** Prediction of endpoints through Cox´s regression models

Characteristics		Odds ratio	95% confidence interval	Standard error	Z-value	*P*-value
All asbestos-related endpoints*						
Sex - female (male as ref.)		1.076	0.759–1.524	0.178	0.410	0.682
Age		0.828	0.807–0.850	0.013	-14.100	< 0.001
Exposure duration		0.982	0.967–0.998	0.008	-2.198	0.028
Dust level - high (low as ref.)		0.648	0.385–1.090	0.265	-1.635	0.102
Cumulative exposure - substantial (mild as ref.)		1.890	1.180–3.023	0.240	2.647	0.008
Place of exposure - Plant B (Plant A as ref.)		1.063	0.800-1.412	0.145	0.420	0.675
Smoking status (non-smoker as ref.)	smoker	0.872	0.614–1.237	0.179	-0.770	0.441
	ex-smoker	1.124	0.790–1.598	0.180	0.648	0.517
Spirometry (normal as ref.)	mild	1.350	1.001–1.820	0.153	1.965	0.049
	moderate	1.523	0.847–2.741	0.300	1.405	0.160
	severe	1.928	1.007–3.693	0.332	1.980	0.048
Symptoms - present (absent as ref.)		1.399	1.035–1.891	0.154	2.183	0.029
Minor radiological findings only*						
Sex - female (male as ref.)		1.137	0.760–1.703	0.206	0.625	0.532
Age		0.823	0.797–0.850	0.016	-11.815	< 0.001
Exposure duration		0.969	0.950–0.988	0.100	-3.144	0.002
Dust level - high (low as ref.)		0.660	0.379–1.150	0.283	-1.467	0.142
Cumulative exposure - substantial (mild as ref.)		1.983	1.175–3.345	0.267	2.566	0.010
Place of exposure - Plant B (Plant A as ref.)		1.002	0.718–1.397	0.170	0.009	0.992
Smoking status (non-smoker as ref.)	smoker	0.852	0.568–1.277	0.207	-0.776	0.438
	ex-smoker	1.132	0.737–1.739	0.219	0.567	0.571
Spirometry (normal as ref.)	mild	1.017	0.705–1.466	0.187	0.089	0.929
	moderate	1.074	0.472–2.447	0.420	0.170	0.865
	severe	1.057	0.368–3.039	0.539	0.103	0.918
Symptoms - present (absent as ref.)		0.878	0.620–1.244	0.177	-0.731	0.465

*Denotes the response (dependent) variable in the respective regression model

**Fig. 1:**
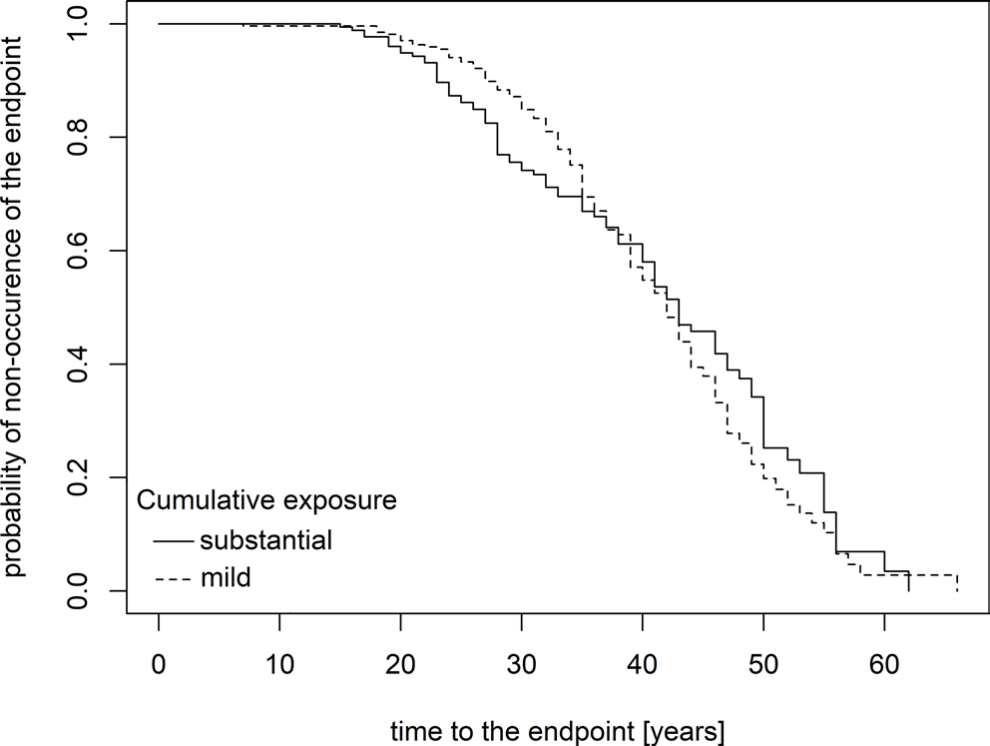
Probability of endpoint-free survival over time (years) since the onset of asbestos exposure, stratified by cumulative exposure levels, for all observed endpoints, including asbestos-related diseases and minor radiological findings

**Fig. 2:**
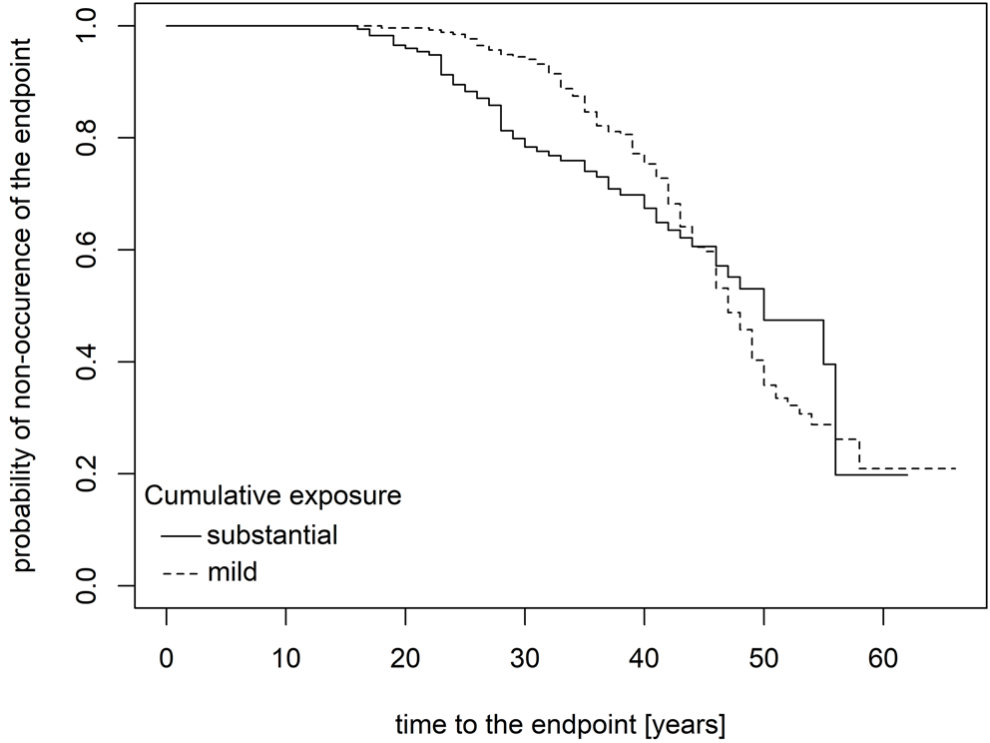
Probability of remaining free of minor radiological findings (pleural and parenchymal lung findings) over time (years) since the onset of asbestos exposure, stratified by cumulative exposure levels

Kaplan-Meier curves (Figs. 1 and 2) demonstrate that the impact of cumulative exposure on the incidence of endpoints became apparent approximately 15 years after initial exposure. In individuals with substantial cumulative exposure compared to those with mild exposure, the decline in the proportion of endpoint-free participants was steeper, particularly for the prediction of minimal radiological changes alone. The halftime to reach 50% endpoint-free individuals was around 40 years for any asbestos-related disorder and 45 years for minimal radiological changes, indicating the earlier onset of diseases in the observed population. For longer latency periods, the curves indicated a less pronounced decline in the proportion of endpoint-free participants among those with substantial cumulative exposure compared to those with mild exposure.

## Discussion

In a sample of individuals with former occupational asbestos exposure, a significant association was observed between cumulative exposure and the subsequent development of pleuropulmonary abnormalities with radiological correlates. Descriptive statistics (Table 1) revealed a gradient in substantial cumulative exposure, highest among individuals with asbestos-related diseases, lower among those with minor radiological findings, and lowest among those without any observed endpoints. Similarly, Cox regression analysis confirmed cumulative exposure as a strong predictor of outcomes (almost double the odds of endpoint in case of substantial exposure). While dust levels did not differ significantly across subgroups, exposure duration exhibited a gradient, increasing from individuals without endpoints to those with asbestos-related diseases. Notably, this trend contrasted with the inverse relationship between exposure duration and outcomes detected in the regression models. This discrepancy may have arisen because descriptive analysis reflected uncensored data (a “best-case scenario”). In contrast, Cox regression accounted for right-censored observations (Bender et al. 2005; Kleinbaum et al. 2012), such as individuals still under follow-up (43.6%) or those whose follow-up ended for reasons other than fatal asbestos-related disease. Despite this, cumulative exposure, as a composite variable incorporating both duration and dust level, consistently emerged as a robust predictor of minor radiological findings and more severe asbestos-associated conditions in both descriptive and regression analyses.

Numerous studies have confirmed a causal link between asbestos exposure and multiple adverse health outcomes, including mesothelioma, lung cancer, hyalinosis, asbestosis, and other cancers (IARC 2012; Loomis et al. 2019). An Italian pooled cohort study on long-term mortality in exposed workers (*n* = 51,801) demonstrated a statistically significant increase in pleural malignancies depending on cumulative exposure (expressed in fibre-years) in both sexes. Notably, non-malignant chest changes also exhibited this dependence, predominantly appearing in the second and third tertiles of cumulative exposure. Furthermore, a statistically significant excess was observed in cancers of the lip, oral cavity, and pharynx with their incidence increasing across tertiles of cumulative exposure (Magnani et al. 2020). Cumulative asbestos exposure of 25 fibre-years (25 fibres/mL/year) is associated with the onset of asbestosis and approximately doubles the lung cancer risk relative to nonexposed individuals, particularly for mixed fibre types (Klebe et al. 2019). In the current study, substantial cumulative exposure nearly doubled the risk of pleuropulmonary abnormalities. Precise fibre concentration data were unavailable for some participants, as measurements were conducted primarily during occupational disease assessments or workplace inspections by public health authorities, rather than through a systematic monitoring framework. Consequently, dust levels for certain individuals were estimated based on occupational roles and comparable exposure conditions.

Existing evidence suggests that asbestos may also be linked to cardiovascular and autoimmune diseases, further underscoring the need for research extending beyond pleuropulmonary effects to encompass other organ systems (Moitra et al. 2023; Pfau et al. 2014).

The recognition of occupational diseases varies by country. In the Czech Republic, lung, laryngeal, or ovarian cancer can be recognised as an asbestos-related occupational disease only when linked to asbestosis or hyalinosis, with verified workplace asbestos exposure as part of a hygiene survey (Government Reg. No. 290/1995). For example, in Germany, cumulative asbestos exposure of ≥ 25 fibre-years suffices for recognition of lung or laryngeal cancer, even without chest abnormalities (Baur 2018). Our study confirmed cumulative exposure as a key predictor of pleuropulmonary diseases and abnormalities.

PPs are localized areas of fibrous thickening, typically affecting the parietal and diaphragmatic pleura. They usually appear as white or white-tan lesions with a rubber-like consistency but may harden due to diffuse calcification. PP is the most common disorder associated with asbestos exposure and serves as a marker of such exposure (Maxim et al. 2015). In the present study, PP was also the most frequently detected radiological finding. Although benign, PP warrants attention due to its reported association with an increased risk of pleural mesothelioma (hazard ratio [HR] 6.80) (Pairon et al. 2013) and lung cancer (HR 3.13) (Gallet et al. 2022). However, other studies have not established a link between PP and lung cancer (Brims et al. 2020). Despite this plausible association with malignancies, PP is not considered an independent risk factor (Maxim et al. 2015).

In a study by Menant et al. (2024) among 5,392 retired workers occupationally exposed to asbestos in France, the risk of PP grew with increasing cumulative exposure to asbestos adjusted for time since first exposure, age and smoking status. Notably, our results demonstrated a stronger association of cumulative exposure with minor radiological findings than with asbestos-related diseases (OR 1.98 vs. 1.89). Similar to our approach, Menant et al. did not rely on precise dust concentration details for their calculations, as metrology data from the time of exposure were unavailable or atmospheric measurements had not been performed. Instead, they employed a cumulative exposure index in each subject over his working life as the sum of exposures calculated for each exposed job (duration × four weighting factors of exposure intensity). Our study narrowed dust level and exposure duration to binary variables to determine cumulative exposure. This limitation, inherent to the significant time lapse since historical exposures, complicates comparisons between similar studies.

Reaching a correct diagnosis of PP requires a good knowledge of normal locoregional anatomy and a rigorous technical approach to chest imaging examination. The patient’s job history should always be kept in mind (Mazzei et al. 2017). Most frequently, PPs are diagnosed using radiographic methods including chest X-ray or CT. CT scans increase sensitivity and specificity compared to standard X-rays (Maxim et al. 2015), consistent with the higher detection rate of PPs in the subgroup of minor radiological changes by (PET)/CT compared to X-rays in the present study.

Typically, asbestos-induced diseases show a long latency period between the first exposure and the disease diagnosis, often occurring years after the exposure has ceased (Öner et al. 2020). Asbestos-related diseases peaked after a mean latency period of 38 years in Germany (Baur 2018), as in the present sample (Table 1). The latency period for PP is typically reported as 15–40 years (Maxim et al. 2015). In the current study, based on Kaplan-Meier curves, the onset of isolated minor radiological changes appeared to be delayed by several years compared to the onset of asbestos-related diseases.

All asbestos types are carcinogenic, with crocidolite exhibiting a higher malignancy potential than chrysotile (IARC 2012). Asbestos fibres, particularly amphiboles, induce chronic inflammation via inflammasome activation, triggering the release of proinflammatory cytokines. Reactive oxygen species contribute to this process, increasing the risk of inflammation-mediated diseases like mesothelioma, pleural diseases, fibrosis, and lung cancer (Cox 2018). Epidemiological data show higher mesothelioma incidence in crocidolite-exposed cohorts (Beckett et al. 2023). In this study, endpoint analysis—covering malignant, non-malignant, and minor radiological findings—showed no significant association between asbestos type (chrysotile and crocidolite in Plant A vs. chrysotile-only in Plant B) and endpoint occurrence (Tables 1 and 2). This was likely to reflect the composite endpoint approach, which emphasized shared pleuropulmonary abnormalities rather than separating malignancies given the common pathophysiological background.

This study has several limitations. Precise asbestos dust concentration data were unavailable for some participants, as workplace dust levels were assessed intermittently during occupational hygiene investigations and did not comprehensively cover all individuals and periods. Cohort heterogeneity is another limitation, as some participants were included through routine post-exposure monitoring while others entered due to suspected occupational diseases. Consequently, selection bias may have occurred, as the study also included referral-based patients, meaning the sample may not be fully representative of the overall workforce in both plants. Additionally, uncertainty arises from potential right-censoring, since reasons for terminating follow-up were not systematically documented. Despite these limitations, the study’s strength lies in its longitudinal design, with participants undergoing regular evaluations usually over several decades, providing robust data on the long-term impacts of occupational asbestos exposure. Additionally, the study population included workers exposed to different types of asbestos. These aspects enhance the study’s contribution to understanding the full spectrum of asbestos-related pleuropulmonary changes.

## Conclusion

Cumulative asbestos exposure emerged as a significant predictor of both asbestos-related diseases and minor radiological findings, irrespective of the specific type of asbestos processed. The findings underscore the importance of continued monitoring for individuals with former occupational asbestos exposure, even those presenting only with minor radiological changes, as these may indicate substantial past exposure and an elevated risk for future severe conditions. Despite regulatory bans, the pervasive presence of asbestos in older buildings poses ongoing risks, highlighting the need for vigilance during renovations and demolition activities. This study reinforces the necessity of proactive post-exposure surveillance programs and stringent occupational health policies to mitigate the long-term impact of asbestos exposure.

## Data Availability

The datasets used and analysed during the current study are available from the corresponding author on reasonable request.
